# Assessing the burden of treatment-emergent adverse events associated with atypical antipsychotic medications

**DOI:** 10.1186/s12888-017-1213-6

**Published:** 2017-02-13

**Authors:** Pierre-Michel Llorca, Christophe Lançon, Ann Hartry, T. Michelle Brown, Dana B. DiBenedetti, Siddhesh A. Kamat, Clément François

**Affiliations:** 1 CMP B CHU Clermont-Ferrand, Université Clermont-Auvergne, Clermont Ferrand, Cedex 1 France; 2 Laboratoire de Santé Publique Évaluation des Systèms de Soins et Santé Perçue, Université de la Méditerranée, Marseille 5, France; 3 Lundbeck, Deerfield, IL USA; 4 RTI Health Solutions, Research Triangle Park, Charlotte, NC USA; 5 Otsuka Pharmaceutical Development Corporation, Princeton, NJ USA

**Keywords:** Schizophrenia, Major depressive disorder, Atypical antipsychotics, Treatment-emergent adverse events

## Abstract

**Background:**

Treatment of schizophrenia and major depressive disorder (MDD) with atypical antipsychotics (AAPs) show improved efficacy and reduced side effect burden compared with older antipsychotic medications. However, a risk of treatment-emergent adverse events (TEAEs) remains. TEAEs are hard to quantify and perspectives on the importance of TEAEs differ across patients and between patients and physicians. The current study is a qualitative assessment that investigates TEAEs of AAPs from both patient and physician perspectives to provide better understanding of the occurrence and burden of TEAEs associated with these medications.

**Methods:**

Focus groups comprised of patients with MDD and interviews with patients with schizophrenia were conducted at two qualitative research facilities, along with a physician focus group at one of the facilities. Information collected from patients included an exhaustive list of TEAEs experienced, and the frequency and level of bother of each TEAE; from psychiatrists, information included an exhaustive list of TEAEs based on personal observations and patient report, frequency of TEAEs, clinically important TEAEs, and levels of patient-perceived bother. Standard qualitative analysis methods were used to identify, quantify, characterize, and summarize patterns found in the data collected.

**Results:**

A total of 42 patients (25 with MDD and 17 with schizophrenia) and 4 psychiatrists participated in the study. TEAEs reported as bothersome across both patients groups included cognitive issues, weight gain and/or increased appetite, low energy, extrapyramidal symptoms (EPS), and need to sleep/excessive sleep/excessive sleepiness. TEAEs considered more bothersome by patients with schizophrenia were weight gain, low energy, EPS, mental anxiety, and increased positive symptoms; those considered more bothersome by patients with MDD were cognitive issues, somnolence/sedation, and flat/restricted affect. TEAEs considered most clinically important by psychiatrists included metabolic syndrome, weight gain, neutropenia, hyperglycemia, and QT prolongation; those TEAEs considered most bothersome to patients from physicians’ perspectives included weight gain, reduced sexual desire or performance, EPS, akathisia, and hormonal issues.

**Conclusions:**

The wide range of TEAEs that are both frequent and bothersome and the variation in perceived burden according to diagnosis highlight the need for a tailored TEAE-awareness approach when choosing an AAP.

## Background

Atypical antipsychotics (AAPs) are an effective treatment for many types of mental illnesses. According to treatment guidelines, antipsychotics are recommended for schizophrenia treatment [1–3], for the treatment of bipolar disorders [4, 5], and in some cases, as adjunct therapy for major depressive disorder (MDD) [6, 7]. Although effective, AAPs are often associated with treatment-emergent adverse events (TEAEs), which can be highly burdensome and can affect quality of life and medication adherence [8–10]. Accordingly, treatment guidelines recommend that physicians modify treatment regimens based on patients’ response and ability to tolerate side effects [1–7]. This report focused on schizophrenia and MDD as two groups that could be anticipated to experience the effects of the medication very differently, as it was important to study the scope of how patients experience TEAEs. In recent decades, AAPs have been introduced for the treatment of schizophrenia and MDD, with both improved treatment efficacy and reduced neurological side effect burden compared with older, first generation antipsychotics [11, 12]. Schizophrenia affected an estimated 1.1% of adults (2.6 million) in the United States in 2013, and onset in early adulthood is common, often leading to chronic lifelong disability [13, 14]. MDD is even more prevalent in the United States, with an estimated 6.7% (15.7 million) adults having experienced an MDD episode in 2013, and more than 10 million who received treatment for depression [15]. However, despite the improved efficacy and tolerability profiles of AAPs, the risk of TEAEs still associated with these agents often includes weight gain and metabolic syndrome, extrapyramidal symptoms (EPS), sexual dysfunction, and sedation and somnolence, depending on the specific agent [9, 16, 17]. A multiple treatment meta-analysis of schizophrenia trials has shown that although antipsychotics had small but robust differences in efficacy, they differed substantially in side effects [12].

Perspectives on the importance of TEAEs differ across patients and between patients and physicians. That is, how patients are affected by TEAEs is specific to each individual and may also be seen differently by physicians. These differences in perspectives and preferences must be taken into account within the therapeutic alliance, as they may impact treatment decisions when considering the overall benefit-risk profile. In fact, increasing importance is being placed on bringing the patients’ perspectives to the evaluation of the overall benefit-risk profile for treatment [18]. In a study reviewing adverse events of antipsychotics as outcome measures, it was concluded that a patient’s subjective experience of medication should be given more consideration [19]. Although TEAEs are an important consideration for treatment, they are hard to quantify. The patient’s perspective may assist in this and has been used in other disease fields. For example, in rheumatology, a tolerability index that has been used in clinical trials incorporates a patient-based method of assessing TEAEs [20]. In cancer clinical trials, a recommended core set of patient-reported symptoms for measuring side effects has been established to promote consistent assessment of treatment-related symptoms [21]. There are a number of neuroleptic side-effect assessment scales available [22, 23], and among the most complete are the 48-item Udvalg for Kliniske Undersogelser (UKU) rating scale [24] and the Liverpool University Neuroleptic Side-Effect Rating Scale (LUNSERS) [25]. However, these scales do not fully account for the patient’s subjective experience or preference. Although considered important, the differences in patient and physician perspective in schizophrenia and MDD appear to be lacking in the literature.

The current study investigates TEAEs of AAPs from both patient and physician perspectives. The goal of the study was to provide better understanding of the occurrence and burden of TEAEs associated with AAP medications, as reported by patients and physicians. The initial feedback gained in this study will be used for a future project to generate an algorithm for a tolerability index score that will quantify the burden of AAP TEAEs and fully accommodate patient preference through a discrete choice experiment. The development of this index measure to assess the burden of TEAEs could help facilitate the prescribing of AAP medications to individuals with MDD and schizophrenia. A first step to establishing this potential tolerability index algorithm would be to evaluate which TEAEs are most bothersome to patients. Although the long-term goal is to allow the approach towards assessing TEAEs to be transnational, the patient selection was based in the US, partly due to the push by the FDA to include patients’ perspectives in the overall benefit-risk profile for treatment [18].

## Methods

### Study design

To gain insight into the occurrence and burden of TEAEs associated with AAPs, focus groups with patients with MDD and interviews with patients with schizophrenia were conducted at two qualitative research facilities: one in Raleigh, North Carolina, USA, and one in St. Louis, Missouri, USA. Although focus groups are more efficient in terms of the time required for data collection and appropriate for data collection in the MDD patient population, previous studies have demonstrated that individual interviews are more successful in obtaining adequate feedback from individuals with schizophrenia, as these patients are generally more comfortable discussing their symptoms and experiences on an individual basis [26]. A physician focus group was also conducted with psychiatrists at the North Carolina location to obtain their perspectives on the occurrence and importance of TEAEs in these patient populations. This study fully adhered to COREQ guidelines and methodology. Medical recruiters at each facility screened all participants following a study review by an institutional review board (RTI IRB Approval 2/18/15; # 13733); informed, written consent was obtained prior to initiation of the study. Patients and physicians were provided an honorarium in appreciation for their time.

### Physician and patient recruitment

Patients were selected from the individual site databases of general community residents who had previously agreed to be contacted for potential research opportunities. Patients were identified and screened, based on their own reports, to meet the inclusion criteria of being an English-speaking adult with a clinician-administered diagnosis of MDD or schizophrenia, taking one or more AAPs within the past year, and reporting one or more TEAEs associated with an AAP. The AAPs included were: aripiprazole, asenapine, clozapine, iloperidone, lurasidone, olanzapine, paliperidone, quetiapine, risperidone, or ziprasidone. Physicians interested in participating in the study were identified from the North Carolina site database. Each physician was selected by meeting the criteria of being a practicing psychiatrist providing direct care of adult patients with MDD and/or schizophrenia and regular treatment of these patients with AAP medication (e.g., 10% or more of patients with MDD requiring adjunctive therapy).

### Procedures

In this study, a TEAE was defined as “any untoward or undesirable medical occurrence in a patient that was linked in time with the use of a pharmaceutical/medicinal product and that may or may not be considered to be related to that product.” Adverse drug events were not actively solicited, ascertained, or evaluated in the study; however, because this project was conducted by Lundbeck, if a potential TEAE associated with a Lundbeck product became evident through the conduct of this qualitative research, a TEAE report was submitted to Lundbeck US Pharmacovigilance.

Semi-structured interview guides were utilized to provide structure to the MDD focus groups (lasting approximately 1.5 h each), the schizophrenia individual interviews (lasting 45 min each), and the psychiatrist focus group (lasting approximately 1.5 h). All focus groups and interviews were conducted by two PhD-level psychologists. General discussion was followed by targeted questions along with handouts for each participant to provide individualized feedback. The following information was obtained at the group or individual patient level: 1) exhaustive lists of TEAEs experienced, 2) frequency of each TEAE, and 3) bother ranking for the most bothersome TEAEs (“1” for most bothersome TEAE, “2” for the next most bothersome, and so on; up to a number that seemed meaningful to the patient). At the physician level, collected information included: 1) an exhaustive list of TEAEs observed or reported by their patients; 2) the most and least frequently occurring TEAEs; 3) clinically important TEAEs (ranked as 1 for most clinically important); and 4) level of patient-perceived bother for each clinically important TEAE (0 = no bother to 10 = extremely bothered). Physicians were not asked to distinguish between adverse event profiles for patients with MDD and schizophrenia.

### Analysis

To ensure consistency in organizing and coding TEAEs across patients and physicians, a codebook was developed and applied, providing consensus in TEAE coding decisions by each of the two focus group and interview moderators.

In order to organize the rankings and rating scores in a more meaningful manner, a “top 3 box” approach was taken (i.e., collapsing the proportion of participants reporting in the top 3 responses). For example, bother and clinical importance rankings of 1, 2, and 3 were collapsed into the “most” bothersome or “most” clinically-important TEAEs, and ratings of 8, 9, and 10, among physicians in rating patient burden were collapsed into the “most” bothersome to patients. Given the qualitative nature of the study, no formal statistical analyses or comparisons were conducted.

## Results

### Patients and physicians

A total of 42 patients participated in the study—25 patients with MDD and 17 with schizophrenia (Table 1). More than half of the patients with MDD were female (64%) and more than half of the patients with schizophrenia were male (65%). The most common currently prescribed AAPs in patients with MDD were quetiapine and aripiprazole (both 24%) and for patients with schizophrenia, risperidone and olanzapine (both 24%). These AAPs were also the most commonly prescribed to each of the two patient groups within the last year. Of patients with MDD, 44% were diagnosed within the last 10 years while 35% of patients with schizophrenia were diagnosed within that time frame. Of patients with MDD, 48% reported living with a spouse or partner while only 4% reported living with roommates who were not family members. Patients with schizophrenia more often reported living with non-family roommates (29%) than living with a spouse or partner (24%).

**Table 1:** Patient characteristics at screening

Characteristic	MDD (*n* = 25)	Schizophrenia (*n* = 17)	Total Patients (*N* = 42)
Sex, *n* (%)			
Male	9 (36)	11 (65)	20 (48)
Female	16 (64)	6 (35)	22 (52)
Age, years			
Mean (range)	46.4 (23–70)	45.5 (25–59)	46.0 (23–70)
Race/ethnicity,^a^ *n* (%)			
White	20 (80)	9 (53)	29 (69)
African American	3 (12)	8 (47)	11 (26)
Asian	1 (4)	0 (0)	1 (2)
Hispanic/Latino	1 (4)	1 (6)	2 (5)
Mixed race	0 (0)	1 (6)	1 (2)
Current atypical medications, *n* (%)^b^			
Quetiapine	6 (24)	2 (12)	8 (19)
Aripiprazole	6 (24)	2 (12)	8 (19)
Risperidone	3 (12)	4 (24)	7 (17)
Olanzapine	0 (0)	4 (24)	4 (10)
Lurasidone	3 (12)	2 (12)	5 (12)
Clozapine	1 (4)	2 (12)	3 (7)
Ziprasidone	2 (8)	1 (6)	3 (7)
Paliperidone	0 (0)	1 (6)	1 (2)
Fluphenazine	0 (0)	1 (6)	1 (2)
Asenapine	0 (0)	1 (6)	1 (2)

^a^ Participants could report more than 1 race

^b^ Total may not equal 100%. Four patients with schizophrenia and one patient with MDD reported using more than one AAP, and four patients with MDD and 2 patients with schizophrenia had a recent history of AAP use but were not currently using an AAP at screening. Each medication listed may include reference to 1 or more brand names, the chemical or generic name, and/or different formulations

Four psychiatrists participated in the study; all were male and they had an average of 21.5 years of experience in practice. In total during the last year, the psychiatrists treated approximately 600 patients with MDD (35% of whom used AAPs) and approximately 300 patients with schizophrenia (57% of whom used AAPs). All of the psychiatrists regularly prescribed quetiapine, aripiprazole, risperidone, olanzapine, lurasidone, and ziprasidone; 75% of them regularly prescribed clozapine and paliperidone; and 50.0% regularly prescribed asenapine.

### Patient results

During the MDD group discussions and the schizophrenia interviews, exhaustive lists of TEAEs were developed. The first step was to gather a list of AEs from the patients via spontaneous elicitation, followed by queries to the patients from a target listing of TEAEs. Specific TEAEs reported by more than half of patients overall (across both patient types) included: weight gain (76%), cognitive issues including decreased ability to attend, concentrate, remember, or recall (79%), need to sleep/excessive sleep/excessive sleepiness (71%), low energy (67%), EPS (62%), and anxiety (55%) (Tables 2 and 3). The reporting rates of the TEAEs differed between the two groups of patients. For example, patients with MDD were most likely to report cognitive issues as a TEAE (92%) while patients with schizophrenia were most likely to report weight gain as a TEAE (94%). Patients with MDD also commonly reported somnolence (76%), weight gain (64%), low energy (56%), and EPS (52%). Along with weight gain, patients with schizophrenia commonly reported low energy (82%), EPS (77%), somnolence/sedation (71%), and anxiety (65%).

**Table 2:** Frequency, bother, and most bothersome atypical antipsychotic AEs reported by patients with MDD

	MDD, *n* (%) (*n* = 25)		
AE Categories	Frequency	Bother	Most Bothersome^a^
**Cognitive issues**	**23 (92)**	**18 (72)**	**13 (52)**
Weight changes	20 (80)	–	–
**Weight gain and/or increased appetite**	**16 (64)**	**11 (44)**	**8 (32)**
Weight loss and/or decreased appetite	5 (20)	0	0
Somnolence/sedation	19 (76)	–	–
**Need to sleep/excessive sleep/excessive sleepiness**	**19 (76)**	**9 (36)**	**7 (28)**
Zombie-like/out of it	5 (20)	2 (8)	1 (4)
**Low energy**	**14 (56)**	**8 (32)**	**7 (28)**
EPS	13 (52)	5 (20)	3 (12)
Anxiety^b^	12 (48)	6 (24)	4 (16)
Mental anxiety	6 (24)	2 (8)	2 (8)
Physical anxiety	8 (32)	1 (4)	0
Social anxiety	0	0	0
Sexual function	12 (48)	–	–
Increased sexual desire/activities	1 (4)	1 (4)	0
Reduced sexual desire or performance	11 (44)	5 (20)	2 (8)
Anticholinergic-related dryness	12 (48)	–	–
Dry eyes	3 (12)	0	0
Dry mouth	9 (36)	3 (12)	1 (4)
Dry skin	2 (8)	2 (8)	0
Disequilibrium	11 (44)	3 (12)	0
Insomnia	9 (36)	3 (12)	2 (8)
Restlessness/akathisia	9 (36)	5 (20)	2 (8)
**Flat/restricted affect**	**8 (32)**	**7 (28)**	**6 (24)**
Anger/aggression	8 (32)	5 (20)	3 (12)
Irritability	7 (28)	6 (24)	4 (16)
Depressive symptoms	6 (24)	6 (24)	4 (16)
Hypomania	5 (20)	4 (16)	2 (8)
Social withdrawal	5 (20)	2 (8)	2 (8)
Bowel/digestive system changes	5 (20)	–	–
Constipation	1 (4)	1 (4)	0
Diarrhea	3 (12)	1 (4)	0
Nausea/vomiting	2 (8)	0	0
Cardiovascular	4 (16)	–	–
Hypertension	2 (8)	1 (4)	0
Hypotension	1 (4)	0	0
QT prolongation/skipped heartbeat	1 (4)	1 (4)	
Visual problems	3 (12)	2 (8)	0
Abnormal blood/laboratory test levels	3 (12)	–	–
Anemia	0	0	0
Hyperglycemia	1 (4)	1 (4)	1 (4)
Hyperlipidemia	2 (8)	2 (8)	0
Hormonal	2 (8)	2 (8)	0
Pain	2 (8)	1 (4)	1 (4)
Other ^c^	2 (8)	1 (4)	0
Major medical (hypothyroidism)	2 (8)	0	0
Major medical (diabetes)	0	0	0
Major medical (seizures)	0	0	0
Increased schizophrenia positive symptoms	0	0	0

*Abbreviations*: *EPS* extrapyramidal symptoms, *MDD* major depressive disorder, *QT* time between the start of the Q wave and the end of the T wave in the heart’s electrical cycle

^a^ The top 5 AEs most frequently reported as most bothersome are highlighted in bold

^b^ Anxiety is reported as the sum of the participants who endorsed any of the anxiety subcodes and also accounts for participants who reported “anxiety” without noting a subcode

^c^ The category of “other” was created to represent select symptoms reported by 2 or fewer participants that were also not reported as most bothersome, most clinically important, or an AE of specific interest

**Table 3:** Frequency, bother, and most bothersome atypical antipsychotic AEs reported by patients with schizophrenia

	Schizophrenia, *n* (%) (*n* = 17)		
AE Categories	Frequency	Bother	Most Bothersome^a^
Weight changes	16 (94)	–	–
**Weight gain and/or increased appetite**	**16 (94)**	**12 (71)**	**7 (41)**
Weight loss and/or decreased appetite	4 (24)	0	0
**Low energy**	**14 (82)**	**12 (71)**	**6 (35)**
EPS	13 (77)	10 (59)	2 (12)
Somnolence/sedation	12 (71)	–	–
Need to sleep/excessive sleep/excessive sleepiness	11 (65)	6 (35)	1 (6)
”Zombie-like”/”out of it”	4 (24)	2 (12)	1 (6)
**Anxiety** ^**b**^	**11 (65)**	**8 (47)**	**6 (35)**
**Mental anxiety**	**7 (41)**	**6 (35)**	**6 (35)**
Physical anxiety	4 (24)	4 (24)	2 (12)
Social anxiety	1 (6)	0	1 (6)
Cognitive issues	10 (59)	6 (35)	3 (18)
Sexual function	10 (59)	–	–
Increased sexual desire/activities	2 (12)	1 (6)	1 (6)
Reduced sexual desire or performance	8 (47)	4 (24)	2 (12)
**Increased schizophrenia positive symptoms**	**8 (47)**	**7 (41)**	**4 (24)**
Anticholinergic-related dryness	7 (41)	–	–
Dry eyes	1 (6)	0	0
Dry mouth	6 (35)	5 (29)	3 (18)
Dry skin	0	0	0
Visual problems	7 (41)	4 (24)	2 (12)
Insomnia	6 (35)	5 (29)	2 (12)
Restlessness/akathisia	6 (35)	3 (18)	3 (18)
Disequilibrium	5 (29)	3 (18)	1 (6)
Cardiovascular	5 (29)	–	–
Hypertension	4 (24)	1 (6)	1 (6)
Hypotension	1 (6)	1 (6)	1 (6)
QT prolongation/skipped heartbeat	0	0	0
Depressive symptoms	4 (24)	2 (12)	2 (12)
Pain	4 (24)	2 (12)	1 (6)
Bowel/digestive system changes	4 (24)	–	–
Constipation	2 (12)	0	0
Diarrhea	2 (12)	1 (6)	1 (6)
Nausea/vomiting	1 (6)	1 (6)	0
Abnormal blood/laboratory test levels	4 (24)	–	–
Anemia	1 (6)	1 (6)	1 (6)
Hyperglycemia	1 (6)	1 (6)	0
Hyperlipidemia	2 (12)	1 (6)	1 (6)
Irritability	3 (18)	2 (12)	1 (6)
Anger/aggression	2 (12)	2 (12)	1 (6)
Hypomania	2 (12)	2 (12)	0
Hormonal	2 (12)	1 (6)	1 (6)
Other^c^	2 (12)	1 (6)	0
Major medical (diabetes)	2 (12)	1 (6)	1 (6)
Major medical (seizures)	1 (6)	1 (6)	0
Flat/restricted affect	1 (6)	0	0
Social withdrawal	1 (6)	0	0
Major medical (hypothyroidism)	0	0	0

*Abbreviations*: *EPS* extrapyramidal symptoms, *QT* the time between the start of the Q wave and the end of the T wave in the heart’s electrical cycle

^a^ The top 5 AEs most frequently reported as most bothersome are highlighted in bold

^b^ Anxiety is reported as the sum of the participants who endorsed any of the anxiety subcodes and also accounts for participants who reported “anxiety” without noting a subcode

^c^ The category of “other” was created to represent select symptoms reported by 2 or fewer participants that were also not reported as most bothersome, most clinically important, or an AE of specific interest

Using the exhaustive list of TEAEs generated in each group or interview as a reference guide, patients reported those that they perceived as bothersome and, of those, further delineated the TEAEs they found to be “most bothersome” through a ranking process. Table 2 lists the frequencies of TEAEs reported by patients with MDD as well as those identified as bothersome and the bothersome TEAEs ranked as the top 3 “most bothersome”. Table 3 lists the frequencies of TEAEs reported by patients with schizophrenia. The table includes the TEAEs identified as bothersome, as well as those ranked in the top 3 “most bothersome.” Specific TEAEs reported as bothersome across the overall patient sample (across both patient groups) included cognitive issues (57%), weight gain and/or increased appetite (55%), low energy (48%), EPS (36%), and need to sleep/excessive sleep/excessive sleepiness (36%). Again, the pattern of results differed between the two patient groups. Patients with MDD were most likely to include cognitive issues, weight gain, and excessive sleepiness as bothersome issues (72, 44, and 36%, respectively). These same three TEAEs were also most likely to be selected as most bothersome by patients with MDD (52, 32, and 28% of patients, respectively) although the TEAE of low energy was also rated as most bothersome 28% of the time. In contrast, patients with schizophrenia were most likely to include weight gain (71%), low energy (71%), and EPS (59%) on the list of bothersome TEAEs, and then to select weight gain, low energy, and anxiety as the most bothersome symptoms (41, 35, and 35%, respectively).

There were other findings that were common across both patient groups. For instance, reduced sexual desire was mentioned as a TEAE by 44% of patients with MDD and by 47.1% of patients with schizophrenia; however, neither group was likely to endorse this TEAE as the most bothersome (8.0% for MDD and 11.8% for schizophrenia).

The burden or impact of TEAEs varied in the patient groups, although some generalities could be made. For example, cognitive issues were reported by both groups of patients although patients with MDD reported a more significant impact, including trouble holding conversations, trouble managing work or school, concern about “losing it” or having permanent memory problems, and having poor self-esteem resulting from “feeling stupid”. Participants in both groups noted concerns about gaining weight: small amounts gained quickly, and large amounts gained over a longer period. Most patients attributed weight gain to AAP use and patients in both groups noted that the weight gain resulted in physical problems, poor body image, and poor self-esteem. Additionally, whereas patients with schizophrenia verbalized that they would likely discontinue their medications because of significant weight gain and the related concern about the impact on existing and future cardiometabolic TEAEs, patients with MDD noted that they would rather live with the TEAEs associated with the medications than with extreme depressive symptoms. Both groups also noted increased fatigue, low energy, and sleepiness, usually immediately following AAP initiation and, especially in the case of patients with schizophrenia, these TEAEs were noted with each administration of medication. The impact of the somnolence/sedation was significant and similar in the two groups and included missing time with family and friends, missing social activities, lack of energy leading to not eating properly, poor self-esteem, and feelings of sedation that interfered with proper functioning. Patients in both groups reported EPS symptoms, including tremors and irregular jerky movements, but only patients in the schizophrenia group reported them as burdensome and impactful. Impact in these patients included the fear of others noticing tremors in public and interference with job duties. Patients in both groups noted change in sexual desire and functioning, and while most patients who reported this experienced decreased sexual desire effect, a few patients experienced increased desire. However, in both cases, most patients did not report an impact.

### Physician results

As a group, the physicians also participated in the creation of an exhaustive list of TEAEs. Using the exhaustive list as an index, the physicians reported and then rated or ranked both clinically important and bothersome TEAEs (Table 4). Those considered most clinically important by at least 2 of the 4 psychiatrists were metabolic syndrome (100%), weight gain (50%), neutropenia (75%), hyperglycemia (50%), and QT prolongation (50%). TEAEs considered most bothersome to patients by at least 2 of the 4 psychiatrists were weight gain (100%), reduced sexual desire or performance (50%), EPS (50%), akathisia (50%), and hormonal issues (50%).

**Table 4:** Atypical antipsychotic AEs reported by physicians as clinically important and/or bothersome

AEs, *n* (%)	Clinically important (*n* = 4)	Most clinically important (*n* = 4)	Most bothersome to patients (*n* = 4)
Metabolic syndrome ^a^	4 (100)	4 (100)	n/a
Weight gain	4 (100)	2 (50)	4 (100)
Reduced sexual desire or performance	4 (100)	1 (25)	2 (50)
Neutropenia ^b^	3 (75)	3 (75)	1 (25)
Hyperglycemia ^b^	3 (75)	2 (50)	0
EPS ^b^	3 (75)	1 (25)	2 (50)
Hyperlipidemia	3 (75)	1 (25)	0
Akathisia	3 (75)	0	2 (50)
QT prolongation ^b^	2 (50)	2 (50)	0
Major medical (seizures)	2 (50)	1 (25)	1 (25)
Hormonal	2 (50)	0	2 (50)
Hypotension	2 (50)	0	1 (25)
Cognitive issues	2 (50)	0	1 (25)
Major medical (diabetes) ^b^	1 (25)	1 (25)	1 (25)
Hypertension	1 (25)	1 (25)	0
Low energy	1 (25)	0	1 (25)
Depressive symptoms	1 (25)	0	0
Flat/restricted affect	1 (25)	0	0
Somnolence/sedation	1 (25)	0	0

*Abbreviations*: *AE* adverse event, *EPS* extrapyramidal symptoms, *QT* time between the start of the Q wave and the end of the T wave in the heart’s electrical cycle

^a^ Reported as “most clinically important” in the group setting but not the individual task

^b^ Reported as “most clinically important” in the group setting and in the individual task

The two TEAEs discussed most by physicians were weight gain and reduced sexual desire or performance. According to the physicians, weight gain was common and almost immediate, but in contrast to patient opinion, it was not always attributed to medication; rather, it was attributed to poor eating habits that were exacerbated during hospitalization or major depressive episode. The physicians believed that the weight gain created a negative impact that was generally more significant for women, but sometimes created a positive impact for individuals with poor appetite associated with MDD, because it helped achieve healthy body weight. Reduced sexual desire or performance was reportedly mentioned within the first visit following medication change or initiation. The physicians commented that it was not always due to AAP, and it was rare to see an improvement of this TEAE while on medication.

### Patient and physician summary

**Fig. 1:**
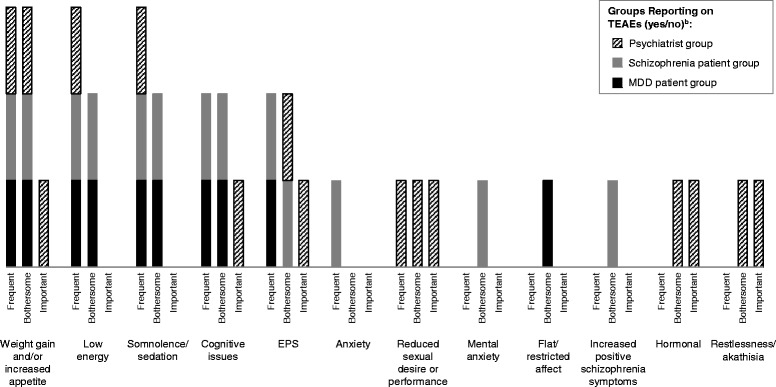
Summary of Frequently Reported, Bothersome, or Clinically Important Adverse Events Reported by Patients and Physicians.^a.^ EPS, extrapyramidal symptoms; MDD, major depressive disorder; TEAE, treatment-emergent adverse event.^a^ “*Frequent*” refers to the overall frequency of report for either patient type or for psychiatrists and refers to TEAEs mentioned by ≥50% of patients or those described as “*frequent*” by psychiatrists; “*Bothersome*” refers to the report of a TEAE as bothersome by ≥30% of either patient type or as “*most*” bothersome by ≥20% of either patient type and for psychiatrists as “*most bothersome*” by 2 of the 4 psychiatrists; “*Important*” refers to TEAEs identified as “*clinically important*” by 2 of the 4 psychiatrists. ^b^ In addition, the following AEs were listed as clinically important by ≥2 physicians: neutropenia, hyperglycemia, hyperlipidemia, QT prolongation, seizures (*major medical*), hypotension, and metabolic syndrome

A summary of frequently reported, bothersome, or clinically important AAP TEAEs conveyed by patients and psychiatrists is illustrated in Fig. 1. This figure shows that patients with MDD and schizophrenia used similar terms to describe their TEAEs and the most frequent and bothersome TEAEs reported by patients were mentioned by the psychiatrists as well. One exception was that the terminology and descriptions of some TEAEs differed between patients and clinicians, such as that of akathisia. Instead of using the clinical term, patients described experiences consistent with akathisia, including “very uncomfortable and shaking inside,” “can’t get comfortable,” “needing to move,” “can’t sit still,” “had to fidget,” and “jumping out of my skin.” Many TEAEs that were highly bothersome were also noted as frequently occurring, including weight gain, low energy, somnolence, cognitive issues, and EPS. TEAEs considered highly bothersome by patients and clinically significant by psychiatrists included weight gain and cognitive issues (MDD and schizophrenia) and EPS (schizophrenia), although the results indicate that cognitive issues are more bothersome than physicians think, especially for MDD patients. In contrast, many other TEAEs were considered clinically important by psychiatrists but not by patients, including metabolic syndrome, reduced sexual function, QT prolongation, hormonal changes, akathisia, neutropenia, seizures, and hypotension. It should be noted that some of these TEAEs (e.g., QT prolongation, neutropenia) are not amenable to self-reporting by the patient and therefore, should not necessarily be considered unimportant by the patient. However, although the patients cannot self-report these specific TEAEs, they were discussed as part of the exhaustive list.

## Discussion

Newer, second generation (atypical) antipsychotics demonstrate improved efficacy and reduced side effect burden compared with older, first generation antipsychotic medications [11, 12]. However, a risk of TEAEs, including weight gain, EPS, sexual dysfunction, and somnolence remains [9, 16, 17]. This study supports these previous findings and sheds light on TEAE perspectives and preferences that may impact treatment decisions for patients with schizophrenia or MDD being treated with these newer agents. These differences should be taken into account to improve the therapeutic alliance between patients and physicians.

This study used focus groups for MDD patients, interviews for schizophrenia patients, and focus groups for physicians to form and rank an exhaustive list of TEAEs to show that patients with MDD and schizophrenia generally used similar words to describe the TEAEs they were experiencing. TEAEs reported as bothersome across both patient groups included cognitive issues, weight gain and/or increased appetite, low energy, EPS, and the need to sleep/excessive sleep/excessive sleepiness. TEAEs considered more bothersome by patients with schizophrenia were weight gain, low energy, EPS, mental anxiety and increased positive symptoms, whereas TEAEs considered more bothersome by patients with MDD were cognitive issues, somnolence/sedation, and flat/restricted affect. Patients’ and physicians’ perspectives did not align as well as those across the two patient groups. TEAEs considered most clinically important by psychiatrists included metabolic syndrome, weight gain, neutropenia, hyperglycemia, and QT prolongation; those considered most bothersome to patients from physicians’ perspectives included weight gain, reduced sexual desire or performance, EPS, akathisia, and hormonal issues. Some of the differences between the perspectives of patients and psychiatrists were related to clinical term use. For example, differences in understanding of akathisia may have been due to differences in terminology as well as differences in description and/or experience, which may explain why clinicians saw it as clinically important and patients did not list it as most bothersome. In addition, due to patients’ tendency to combine types of EPS, the TEAEs of tardive dyskinesia, dystonia, and Parkinson symptoms were combined for the purposes of this study. Interestingly, three of the most frequent and bothersome TEAEs reported by patients (low energy, somnolence/sedation, and cognitive issues) were not described by physicians as being clinically important or most bothersome. This may reflect the challenges that physicians have in measuring and recording these events; but it may also reflect an assumption by the physicians that these symptoms are due to the disease while patients ascribe the adverse events to the treatment. This study did not attempt to tease this apart but this question would be useful for further research in support of improving the therapeutic alliance.

The results described in this study provide better understanding of the occurrence and burden of TEAEs associated with AAP medications as reported by patients and physicians. It is also interesting to compare the frequency and burden ratings from this study to the adverse event profiles of the drugs as observed in the clinical trials and described in the approved product information (PI) sheets. A survey of the top five product PIs as listed by the patients in this study (risperidone, olanzapine, lurasidone, aripiprazole and quetiapine) reveals both similarities and differences. No adverse event is mentioned in all five PIs (using the section on Adverse Reactions with MDD or schizophrenia. Akathisia is listed in four of the five PIs, which is reflected in the common mention by psychiatrists. Other frequent and burdensome AEs, such as weight gain or increased appetite, somnolence, and lethargy/fatigue are each mentioned in three of the five PIs as a common adverse event. However, dizziness and constipation are also listed in three of five PIs but are not mentioned as often by the patients or physicians in this study. Conversely, the very common and burdensome AE of cognitive issues is not mentioned in any PI, nor are sexual function changes. The mismatch between labelling and patient and physician experience further emphasizes the need to better incorporate patient perspective into adverse event reporting and evaluation.

Another way to look at efficacy and tolerability of AAPs is head-to-head clinical trial analysis, as has been shown in a recent mixed-treatment comparison analysis that was used to directly compare the efficacy and tolerability of AAPs [27]. While useful, these types of trials do not reveal how the TEAEs affect patients. To fully understand AE burden in utilization of antipsychotics, the patient perspective needs to be taken into account. Patient attitudes toward antipsychotics are important for adherence to and eventual outcome of the antipsychotic treatment. Surveys of patient attitude toward antipsychotics, including tolerability of TEAEs, have shown a better attitude toward second-generation antipsychotics compared with first-generation antipsychotics, although the results are still debatable [28]. A tolerability index would facilitate the evaluation of TEAE burden among patients and would contribute to the process of prescribing appropriate antipsychotics to patients with MDD and schizophrenia. The information gained in the current study on patient perspectives of TEAE burden will assist in the creation of a tolerability index, which may facilitate appropriate prescribing of AAP medications.

An important consideration based on this study is that if the most burdensome side effects for patients can be determined, physicians can better select the right treatment from the choices available. A multiple treatment meta-analysis of randomized, controlled schizophrenia trials compared 15 antipsychotic drugs and placebo in the acute treatment of schizophrenia and found that whereas antipsychotics had small but robust differences in efficacy, they differed substantially in side effects [12]. The findings challenged the routine classification of antipsychotics by first- and second-generation types (ie, typical and atypical types) and suggested that domain hierarchies should be used to help clinicians adapt the choice of an antipsychotic drug to the needs of individual patients [13]. The results of this study demonstrate the variety of the individual needs of patients, and the potential mismatch between patients’ and physicians’ perspectives. In future research, it will be interesting to further examine sources of variability in patient and physician perspective. For instance, there may be international differences in the perception of burden of these common adverse events that should be carefully described and taken into account in selection of medication in other settings.

This was a patient perspective study, and in addition to the general limitations of a study based on patient reports, it is important to recognize other study limitations. For example, the sample size was small and limited to two geographic locations in the US. A US sample was selected in part because of the wider range of AAPs currently approved for the treatment of MDD in the US, and also because of the recent campaign by the FDA to include patients’ perspectives in the overall benefit-risk profile for treatment [18]. Therefore, the results may not be able to be generalized to a larger or transnational patient or physician population. Further, patient reports proved insufficient for concluding valid results on certain queries. In particular, TEAE onset was investigated because patients are more likely to experience TEAEs early in treatment that may or may not resolve as the patient continues with the treatment. Unfortunately, onset was difficult to determine for most patients and varied greatly in the cases where it was able to be determined. Finally, the data quantitation used a “top 3 box approach.” While this approach enabled the intuitive comparison of perspectives across small and varied sample sizes, this method can also cause a loss of precision. Finally, although unlikely to have affected the study outcomes, the study was funded by a pharmaceutical company and the participating patients and physicians received honoraria for their time.

## Conclusion

The wide range of TEAEs that are both frequent and bothersome and the variation in perceived burden according to the diagnosis highlight the need for a tailored TEAE-awareness approach when choosing an AAP. Following feedback from both patients and psychiatrists, the following TEAEs would probably carry the most weight for a tolerability index measure: weight gain and/or increased appetite, low energy, somnolence/sedation, cognitive issues, EPS, and reduced sexual desire or performance. Other TEAEs that were considered clinically important by psychiatrists included abnormal test results or blood values, cardiovascular issues, major medical issues, hormonal changes, and akathisia. Information gained in this study on TEAE burden associated with AAP use will be helpful in the future development of tools for assessing the overall tolerability of these agents.

## Abbreviations

AAPs: Atypical antipsychotics
AE: Adverse event
EPS: Extrapyramidal symptoms
MDD: Major depressive disorder
TEAEs: Treatment-emergent adverse events
